# Religiosity, Well-Being and ‘Slowing Down’ Ageing Damage: A Literature Review

**DOI:** 10.7759/cureus.9910

**Published:** 2020-08-21

**Authors:** Dimitrios Anyfantakis, Emmanouil K Symvoulakis, Christos D Lionis

**Affiliations:** 1 Primary Care, Primary Health Care Centre of Kissamos, Chania, GRC; 2 Social and Family Medicine, School of Medicine, University of Crete, Heraklion, GRC

**Keywords:** religiosity, telomere length, depression, cellular aging

## Abstract

Telomere length (TL) represents an important marker of cellular aging. Its shortening affects human health and longevity by inducing senescence, apoptosis, and oncogenesis. Advanced ageing and negative behavioral and lifestyle factors decrease TL. The relationship between positive psycho-social factors and longer telomeres has given rise to a growing number of research efforts. Among these, religiosity poses a particular interest since it is associated with a wide range of favorable health outcomes. In this direction, recent literature reports, suggest a positive link between religiosity and TL. Underlying mechanisms for this association are not yet clarified.

In this review, we would like to summarize the current knowledge on the link between religiosity and TL. Taking this opportunity, we recall findings from a cohort study in rural Crete, Greece that adds evidence on the discussion of potential psycho-social mediators which some may prevent shortening of TL.

## Introduction and background

Telomeres are nucleotide structures at the end of each chromosome responsible to maintain genomic stability and integrity [1]. Shortening of telomere length (TL) occurs physiologically with aging [2] and is characterized by adverse biological effects including genetic mutations, cellular senescence, and premature death [3]. Negative lifestyle and dietary habits, anxiety, and depression are well-recognized factors associated with TL shortening [3].

The relationship between positive psycho-social factors and longer telomeres has given rise to a growing number of research efforts. Among these, religiosity poses a particular interest since it is associated with a wide range of favorable health outcomes. In this review, we aimed to retrieve articles that summarize the current knowledge on the potential association between psycho-social mediators and TL.

## Review

We conducted a literature search in the PubMed database using the following Medical Subject Heading (commonly known as MeSH) terms: religiosity [All Fields] AND ("telomere" [MeSH Terms] OR "telomere" [All Fields]) AND length [All Fields]. A total of nine articles were identified and assessed for eligibility. From these, two articles were excluded for no relevance [4-10].

An interesting study by Wang et al. in an elderly Muslim Chinese population found that religiosity was positively associated with TL (p<0.05) and lower depression rates [10]. This association was partially mediated by depressive symptoms in the 65 or older age group (p = 0.015) [10].

The term religiosity as a concept and health determinant has received much criticism and it is usually studied jointly with the term of spirituality. The hypothesis of bio-psycho-social protective factors has been studied a decade before in a rural Orthodox population in the village of Spili in Crete [11,12]. The local economy of this area is based on agriculture and animal husbandry. The studied population was homogeneous in terms of traditional family values and religious characteristics [11,12].

This observational study, named the SPILI III study, aimed to explore to what extent religiosity alleviates the negative effects of stress on a variety of biological and psychological markers [5,6]. Remarkably, highly religious participants presented lower prevalence of diabetes (35.1% vs. 2%, p<0.001) with an estimated diabetes risk, fully adjusted odds ratio, 95% CI: 0.91 (0.87-0.94), lower levels of intimae media thickness (1.01±0.101 vs 1.53±0.502 mm, p<0.001) and lower levels of depression [12,13]. Furthermore, multivariate analyses, unadjusted, age-sex only adjusted and fully adjusted (i.e., for age, gender, family status, smoking habits and body mass index) showed that religiosity was inversely associated with the development of hypertension, diabetes mellitus and cardiovascular disease [12,13].

Seeking a potential explanation for these associations, the authors reported that the church is an ‘institutional’ room of spiritual experience exchange that promotes socialization and a sense of belonging to a group [12,13]. This in turn helps people to create feelings of optimism, meaningfulness, and significance in life [12,13]. Furthermore, religiosity is associated with healthy lifestyle practices such as avoidance of alcohol, smoking, and drug abuse (opioids, cocaine, marijuana) [12,13]. Of course, an interesting question that emerges is which ‘religiosity’ protects [12,13]. For example, being spiritual and offering positive feelings and interactions may better lead to fear eradication and internal peace than accepting religious rituals as a lifestyle obligations [12,13]. For this reason, research design should take into consideration not only definitions but also stochastic meanings and spiritual views of living.

An additional mechanism that may explain the mediating effects of religiosity on mental health and cellular aging is the ability of the human organism to maintain homeostasis in response to chronic stressors [14]. Neuro-endocrine and behavioral processes are implied in the maintenance of homeostasis [14]. Therefore, on the basis of the SPILI III findings, we could presume that religiosity could represent a mediating factor that reduces inflammation, oxidative stress, and hypercortisolemia.

Currently, the senescence phenomenon secondary to TL shortening is considered one of the principal issues of ageing process [15]. Therefore, relieving or suppressing the burden of the senescent cells may ameliorate human health status and increase lifespan [15]. In this direction, the discovery of small molecules that act as selective eliminators of senescent cells (senolytics) became highly promising towards delay of ageing. The first molecules to be identified as senolytics in 2015 were dasatinib, a kinase inhibitor in clinical use and quercetin, a common flavonoid [15].

## Conclusions

The emerging field of psycho-social protective factors as well as novel therapeutic agents with seno-therapeutic and protective effects against DNA damage, seems to shape a promising set of research questions that can be tested in synergy through a loop that links psycho-social well being and deceleration of ageing cascades. It also seems to be an important area for potential research in times of coronavirus disease-19 (COVID-19).
